# Cervical musculoskeletal impairments in migraine

**DOI:** 10.1186/s40945-021-00123-0

**Published:** 2021-12-08

**Authors:** Zhiqi Liang, Lucy Thomas, Gwendolen Jull, Julia Treleaven

**Affiliations:** The University of Queensland, School of Health and Rehabilitation Sciences, St Lucia, Qld Australia

## Abstract

**Background:**

Neck pain is common and disabling amongst individuals with migraine. Cervical musculoskeletal interventions are often sought but there is currently no evidence to support such interventions for this population. Improved understanding of how cervical musculoskeletal impairments present in migraine can elucidate neck pain mechanisms and guide clinicians and researchers in the management of patients with migraine and neck pain.

**Main body:**

Migraine hypersensitivity is a major consideration when assessing for cervical impairments as it can aggravate migraine and confound findings. Current evidence of cervical impairments in migraine is limited by disregard for the different underlying causes of neck pain and possible influence of hypersensitivity. Findings of cervical musculoskeletal impairments are mixed within and across studies, indicating that different forms of neck pain are present in migraine. Some migraineurs have neck pain that is part of the migraine symptom complex and therefore exhibit little or no cervical musculoskeletal impairment. Others have a cervical source of neck pain and therefore exhibit a pattern of cervical musculoskeletal impairments akin to that of cervical disorders. The presence of cervical musculoskeletal dysfunction may or may not be related to migraine but knowledge of this is currently lacking which impacts decision making on management. Cervical musculoskeletal interventions may be indicated for migraineurs with identified cervical dysfunction but other factors requiring further clarification include determination of i) patient specific outcomes, ii) impact of co-existing migraine referred neck pain, and iii) potential moderating effects of migraine hypersensitivity on treatment efficacy.

**Conclusions:**

Physiotherapists should seek a combination of cervical impairments through skilful assessment to identify if cervical musculoskeletal dysfunction is present or not in individual patients. The relevance of cervical dysfunction to migraine and influence of co-existing migraine referred neck pain need to be established through detailed evaluation of pain behaviours and further research. Future clinical trials should define expected treatment outcomes and select individuals with cervical musculoskeletal dysfunction when investigating the efficacy of cervical musculoskeletal interventions.

## Background

Neck pain and migraine are both leading causes of disability globally [1], and almost 80% of migraineurs suffer neck pain [2]. The added burden of neck pain [3, 4] may be why many patients with migraine seek treatment of the neck [5, 6]. However, systematic reviews have found very limited evidence to support the use of cervical interventions in these patients [7, 8]. Despite this lack of evidence, a recent study revealed that most migraine patients preferred cervical interventions (manual therapy and exercise) over aerobic exercise in addition to usual migraine treatment [9]. Unfortunately, even recent trials of spinal manipulation [10] and multimodal cervical interventions [9, 11] have failed to demonstrate any benefit for the migraine population. Physiotherapists are therefore faced with a conundrum when patients with migraine and neck pain request cervical interventions.

The clinical dilemma of whether neck treatments should be prescribed for patients with migraine could be addressed by first examining the evidence for cervical musculoskeletal impairments in this population. Cervical interventions offered by physiotherapists are often aimed at addressing specific impairments (e.g., painful joint dysfunction, impaired muscle function), hence accurate identification of such impairments can help direct interventions. More importantly, determining if, and how, cervical musculoskeletal impairments present in individuals with migraine may help discern the underlying mechanisms of neck pain in this population. There is strong theoretical [12], and more recently empirical evidence [13], that neck pain in migraine can originate from two different sources. First, pain could be due to local nociception from cervical musculoskeletal structures, as is the case for cervical musculoskeletal disorders. If so, cervical musculoskeletal impairments should be present and occurring in a combination of impairments similar to that which is found in cervical musculoskeletal disorders [14–17]. On the other hand, pain could be referred from the head into the neck via the trigeminocervical nucleus, which means neck pain is part of migraine symptomology and unrelated to cervical musculoskeletal dysfunction [12]. Impaired musculoskeletal functions are unlikely to be present if neck pain is solely due to migraine. The two mechanisms have different implications for management and could each, or in combination, be the cause of neck pain. Recognising the cause(s) of neck pain in the individual migraineur is crucial for decisions on management. Determining the extent of cervical musculoskeletal impairments present in the individual migraineur will help to identify when there is a cervical origin of neck pain [18].

The aims of this paper are to i) highlight the challenges in the assessment of the neck and in the interpretation of clinical findings in migraineurs, ii) critically interpret the current body of research, iii) discuss the implications of this research, and iv) propose recommendations for clinical practice and future directions for research.

### Challenges in identifying cervical musculoskeletal impairments in migraine

Heightened sensitisation is the hallmark of migraine, and it has untold implications for cervical assessments. Convergence of head and cervical afferents in the trigeminocervical nucleus creates a pathway for migraine sensitisation to spread into the cervical region [19]. This explains why lower cervical pain thresholds are found in many individuals with migraine [20]. Consequently, physical assessments of the cervical spine can be provocative of neck pain and or migraine [13, 21] but are not necessarily indicative of cervical musculoskeletal dysfunction [13]. For example, cervical muscle strength output could be inhibited by pain, without muscle dysfunction [22]. On the other hand, muscle activity may be increased due to trigeminocervical sensitisation [23], which can be misinterpreted as impaired motor control. Increased muscle activity due to stress can occur in migraine but not be associated with the extent of pain experienced during a stressful task [24]. Leistad and colleagues [24] found that some participants with migraine did not report any pain despite recording increases in cervical and cranial muscle activity. In contrast, higher levels of neck pain were reported when slightly reduced muscle activity was recorded. The authors concluded that neck pain and headache during a stressful task was not likely to originate from muscle and was more likely due to stress-induced trigeminocervical sensitisation. Similarly, non-specific effects of physical testing such as concentration or ambient lighting, and collateral effects such as dizziness during head movements, have the potential to confound test results by activating migraine hypersensitivity [12, 13]. Since migraine sensitisation has been recognised to induce a pain response and affect muscle activity, assessment of cervical function should not rely solely on pain responses and muscle activity as outcome measures. Whilst assessments such as palpation of muscle and soft tissues may be clinically valuable to gain information on muscle tone and palpatory tenderness, these positive findings on their own may be due to either cervical dysfunction or sensitisation or a combination of both. For example, reproduction of neck pain and headache from manual palpation of the upper cervical segments [25] may be due to sensitisation mechanisms within the trigeminocervical nucleus causing pain referral between the two regions [26], with or without accompanying cervical musculoskeletal dysfunction. By the same token, assessments of cervical motor control may require other outcome measures besides a measure of muscle activity. To date, there are limited reports on the frequency of symptom aggravation during cervical assessments for the migraine population, with even less investigation into whether symptoms may have confounded results. Improved reporting and evaluation of how to interpret musculoskeletal function when symptoms are produced is needed in research to guide clinicians in their assessment and interpretation of clinical findings in patients who are highly sensitised.

Neuro-mechanosensitivity is another factor to consider when assessing the cervical spine in individuals with migraine. Connective tissues linking the rectus capitis posterior minor muscle to dura mater form a myodural bridge [27] which tractions the dura when the rectus capitis posterior minor muscle is stretched during upper cervical flexion or rotation. Tests that involve these positions, such as the Craniocervical Flexion Test (CCFT) or the cervical Flexion Rotation Test (FRT), may be provocative of symptoms in individuals with heightened neuro-mechanosensitivity. Although the FRT primarily assesses C1–2 motion [28], it is possible that range of motion during the test may be limited by pain without articular restriction in individuals with heightened neuro-mechanosensitivity. Neuro-mechanosensitivity can also provoke symptoms and limit performance of inner range craniocervical flexion during the CCFT. Assessment of neuro mechanosensitivity prior to the performance of these tests is therefore recommended and alternative tests performed if neuro-mechanosensitivity is detected.

Finally, migraine related sensitisation is greatest during and around the time of a migraine episode but can also remain elevated interictally, in between migraine episodes [29, 30]. Hence assessments may be affected at any time. In our recent study, we found that a large proportion of participants with migraine reported neck or headache symptoms during cervical assessments, despite being assessed during interictal periods. Yet, many of them were found to have good overall cervical function, similar to healthy controls [13]. Heightened sensitisation demands that cervical assessments be mindfully chosen, skilfully performed and carefully interpreted in patients with migraine.

### Current evidence of cervical impairments in migraine

There is a large body of research studying cervical impairments in migraine, including two recent systematic reviews [31, 32]. Many cervical measures have been investigated in migraine, encompassing almost every aspect of cervical musculoskeletal function [31, 32]. However, very few studies have reported on the frequency of symptoms experienced by participants during cervical assessment or analysed the impact of sensitisation on their tests results. Furthermore, most studies utilise the traditional research method used in musculoskeletal disorders, which is to compare each musculoskeletal measure across participant groups, i.e., cervical range of motion (ROM) is compared between episodic migraine, chronic migraine, and headache-free groups. This is a major limitation in the current body of research on migraine because some migraineurs with neck pain may have a cervical source of neck pain and therefore present with cervical impairments, but other individuals may not have a cervical source of neck pain and therefore have little or no cervical impairments. When all individuals with migraine are grouped together as a homogenous group, impairments presenting in some individuals may be washed-out by the lack of impairments in other individuals. This may be why results within and across studies are inconsistent, and why only minor impairments are found in meta-analyses [31, 32]. Interpretation of these findings at face value indicates a lack of evidence for cervical impairments in migraine and would therefore oppose the clinical application of interventions targeting cervical impairments. However, these findings may be the result of methodological limitations in previous studies which do not allow for different mechanisms underlying neck pain in individuals. Studies capable of phenotyping individual migraineurs with neck pain are needed to clarify the presence of cervical impairments and guide clinical management.

Our recent study [13] allowed for different mechanisms of neck pain in migraine by comparing the overall outcomes for each participant against that of other participants in the study. We found that migraineurs with neck pain were separated into two distinct groups based on their performance across several cervical musculoskeletal assessments. The migraineurs in one group showed good overall performance in the tests and were indistinguishable from healthy controls, whereas migraineurs in the other group presented with cervical dysfunction related to their neck pain that was comparable to the dysfunction exhibited by participants with idiopathic neck pain, indicating a cervical source of neck pain in this latter group but not in the first group. The findings of this study confirmed that migraineurs with neck pain are not a homogenous group and can be sub-grouped by whether they have cervical musculoskeletal dysfunction or not. Subgrouping by the presence of neck pain is only minimally helpful for identifying cervical dysfunction. As expected, all migraineurs without neck pain in our study had good cervical function, as was found in previous studies that identified migraineurs without neck pain [32, 33]. Our study excluded migraineurs with diagnosed cervical disorders and cervicogenic headache. Despite this, a little less than half of the migraineurs with neck pain in our cohort were found to have cervical musculoskeletal dysfunction and thus may be higher in the general migraine population. However, the presence of neck pain does not necessarily indicate a cervical origin. Even the frequency of headache and neck pain could not differentiate between those migraineurs with and without cervical dysfunction [13]. Subgrouping by episodic or chronic migraine was also not useful for identifying a cervical source of neck pain as there were individuals with episodic or chronic migraine in both groups. This again may be why mixed results were found in previous studies that looked for differences in cervical outcomes between chronic and episodic migraine. While some studies found more cervical impairments in chronic migraine than episodic migraine (ROM [33], FRT [34], extensor muscle activity during the CCFT [35], muscle strength and time to peak force [36]), others have not [37, 38].

In accord with previous studies identifying a cervical source of neck pain in headache [14, 15], cervical dysfunction in our study was also characterised by a combination of movement, articular and neuromuscular impairments. Other aspects of cervical musculoskeletal function not assessed in our study may also be present in the subgroup of migraineurs with cervical dysfunction. These may include other impairments found in cervical musculoskeletal disorders such as changes to muscle cross-sectional area and fibre properties, muscle endurance, and cervical sensorimotor function [39, 40]. However, to date no other study has differentiated between migraineurs with and without a cervical source of neck pain, so it is difficult to interpret findings for other cervical musculoskeletal outcomes. It could be questioned that if no differences were found between migraineurs and healthy controls, could it be due to a wash out effect? Likewise, if any impairments were detected, were the impairments associated with a cervical source of neck pain or secondary to migraine sensitisation? For example, Florencio and colleagues recently found that cervical flexor endurance was reduced in episodic and chronic migraine when compared to healthy controls [41], but the authors were unable to ascertain if the reduction in endurance was due to local muscle impairment or reduced motor cortical drive.

Although no other study has sought a combination of cervical impairments in individual migraineurs, seven studies [14, 37, 38, 42–45] examined at least three different cervical outcomes in migraine cohorts [32]. These studies came to different conclusions. Of these seven studies, no impairments were found in the three studies [14, 42, 43] that minimised heterogeneity in their cohort by excluding cervicogenic headache in their migraine participants. The other studies [37, 38, 44, 45] had mixed findings. Aguila [44] found extension:flexion strength ratio and extension ROM to be reduced but the outcomes of the manual examination, the CCFT and the cross sectional area of cervical extensors were no different from healthy controls. Ferracini et al. [37] detected reduced rotation ROM, high numbers of positive FRT and positive manual examination findings, but no impairments for posture and proprioception. Horwitz and Stewart [45] identified the most impairments (ROM, neural extensibility, pain with muscle stretch and manual examination outcomes), but posture and muscle strength were unimpaired. Methodological issues may have affected this study’s results as range of motion in healthy controls were beyond normal range (mean left rotation = 93.52°) and other results were not fully reported. Similarly, Luedtke et al. [38] reported impairments for CCFT, FRT and manual examination, and no impairments for ROM, posture and the upper cervical quadrant test, but their FRT and CCFT results do not indicate impairment. Unilateral FRT range in healthy controls is expected to be around 44° [46], but mean bilateral FRT range in healthy controls of this study was 98°, while mean bilateral FRT range in migraineurs were 89° and 92° for episodic and chronic migraine respectively. CCFT performance in migraineurs were also within normal range (median of 26 mmHg) [16]. These studies, and the others which have identified differences between healthy controls and migraineurs for individual cervical outcomes, were not designed to recognise if all or only a subgroup of migraineurs exhibit the cervical musculoskeletal impairments.

Overall, our recent findings together with the mixed findings from other studies point to the existence of different forms of neck pain in migraine. On one hand, there are individuals with migraine and neck pain who exhibit a pattern of cervical musculoskeletal impairments indicating cervical musculoskeletal dysfunction. On the other hand, there are migraineurs whose neck pain does not stem from the cervical spine and correspondingly exhibit minor or nil cervical impairments. Differentiation of these groups is essential in future research.

### Implications of cervical impairments in migraine

**Fig. 1:**
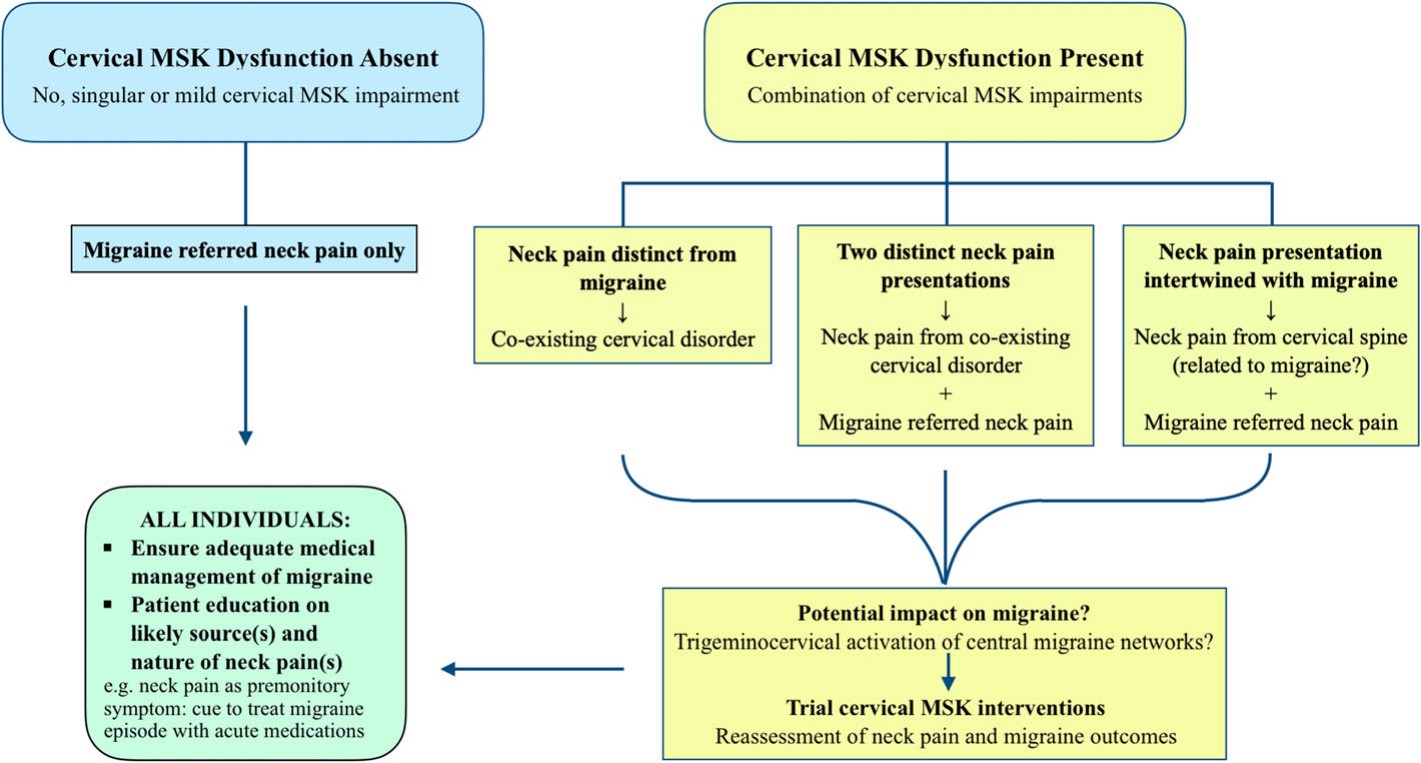
Possible mechanisms of neck pain in migraineurs and implications for management

Cervical impairments could be unrelated to migraine or share a causal relationship that may be bi-directional due to bi-directional sensitisation mechanisms within the trigeminocervical nucleus. It is possible that cervical nociception due to musculoskeletal dysfunction could augment symptoms during migraine episodes, or play a role in triggering migraine attacks by facilitating trigeminocervical sensitisation to activate central migraine networks [47, 48]. Conversely, cervical dysfunction might develop due to disuse or pain inhibition in persistent migraine-referred neck pain. These theories have yet to be proven. The latter may be less likely because our study found no differences in headache or neck pain frequencies between migraineurs with and without cervical dysfunction. Ironically, the individuals without cervical dysfunction had a longer history of neck pain than those with dysfunction. It is also possible that cervical impairments may be unrelated to migraine in some individuals. Anecdotally, a participant in our study reported two distinct versions of neck pain: one that was consistently left-sided and only ever presented after working in awkward neck postures; the other was consistently right-sided and only ever presented with migraine that was also only right-sided. The two neck pains never occurred together, and the left-sided neck pain was never present with migraine. Cervical dysfunction was detected in this participant, but only on the left side. This individual’s presentation indicates two different forms of neck pain which are separate and independent of each other: left-sided neck pain is consistent with cervical musculoskeletal disorder whereas right-sided neck pain stems from migraine. Since this individual never experienced left-sided neck pain with migraine, their cervical impairments were most probably unrelated to migraine. Clinical presentations of patients with migraine and neck pain are often less straightforward than the above scenario. When cervical musculoskeletal dysfunction is present, it could indicate a cervical disorder that is either co-existing or related to migraine. Migraine referred neck pain may also be present alongside neck pain of cervical origin. The complex interplay between cervical dysfunction and migraine is not fully understood and requires further research to guide management (Fig. 1).

Should cervical interventions be prescribed for patients with migraine who have been identified to have cervical dysfunction? This is a question that everyone wants answered, but it needs refinement and deeper investigation. Some of the issues to consider include, first, is the aim of intervention to improve neck pain or migraine or both? Second, although cervical interventions that target specific cervical impairments may seem appropriate to address neck pain of cervical origin, will they have much effect on individuals who have two forms of neck pain, i.e., migraine-referred neck pain is also present? Third, since individuals with migraine are aggravated by cervical assessments [13, 21], will migraine-related factors such as sensitisation limit treatment options and efficacy? These are all issues to be considered by clinicians and researchers. Future clinical trials should clearly define expected outcomes and select migraineurs with neck pain of potentially cervical origin if investigating the effects of interventions commonly used for cervical musculoskeletal disorders.

### Recommendations for clinicians

A wide range of cervical musculoskeletal assessments are available to physiotherapists. Selection of which assessments to perform should be guided by clear aims and the individual patient’s tolerance to assessments. If the aim of examination is to ascertain if there is a cervical source of neck pain, physiotherapists should search for a combination of impairments using assessments that span different domains of cervical musculoskeletal function, i.e., movement, neuromuscular, and articular. Singular positive test findings do not reflect the typical presentation of musculoskeletal disorders and may be secondary to migraine sensitisation. Identified impairments should also relate to the patient’s presenting neck pain, e.g., same side as reported neck pain, or in similar aggravating positions. Prioritisation and skilful implementation of assessments is necessary so as not to overtax the sensitised patient and aggravate symptoms. In particular, tests that require a pain response, such as manual examination, need to be performed and interpreted with the effects of sensitisation in mind. Sensory tests, such as pressure pain thresholds, to ascertain the degree of sensitivity in the individual patient may complement and aid interpretation of musculoskeletal test findings. For instance, the false positives during manual examination may be more likely when accompanied by low cervical pressure pain thresholds. In patients who have been identified to be highly sensitised, interpretation of assessment findings should rely less on pain responses and be focussed on other outcomes. For example, pain responses during the FRT may not be reliable as the sole indicator for determining the limit of C1–2 ROM in patients who are highly sensitised in the upper cervical spine. Other indicators such as the assessor’s perception of end-feel at the onset of pain may be more informative; an absence of firm end-feel may suggest that full available range has not yet been reached. Consideration of non-specific factors may help to minimise fear or stress that could confound test results. These include the explanation or instructions conveyed to patients, the speed at which a passive movement is performed or the testing position.

Individualised patient education based on assessment findings is an essential component of management. This should include the likely source(s) of neck pain and degree of cervical hypersensitivity, as well as the implications for management. In particular, patients need to understand if neck pain is referred from migraine and is a premonitory symptom. This is because when migraineurs fail to recognise neck pain as a premonitory symptom and delay treatment, the efficacy of acute migraine medication is reduced [49]. When cervical musculoskeletal dysfunction is identified in an individual with migraine, physiotherapists still need to determine if migraine also contributes to neck pain. Does the extent of impairments relate to the intensity, frequency and behaviour of neck pain reported by the patient? Physiotherapists also need to identify if there is a relationship between cervical dysfunction and migraine and determine if sensitisation may hinder intervention. Cervical interventions targeting musculoskeletal impairments should only be prescribed for individuals proven to have cervical musculoskeletal dysfunction. Since the efficacy of such interventions is yet to be demonstrated, physiotherapists should engage patients in an open discussion regarding each patient’s expected outcomes. Outcomes need to be reassessed carefully throughout the treatment period and interventions discontinued if meaningful outcomes are not met. Ultimately, it is vital to ensure that appropriate medical management of migraine is in place and that physiotherapy treatment does not aggravate migraine (Fig. 1).

## Conclusions

There are different forms of neck pain that can be independent or coexist in individuals with migraine. Correspondingly, migraineurs who have a cervical source of neck pain present with cervical musculoskeletal impairments but those whose neck pain stems solely from migraine do not. Assessments of cervical musculoskeletal function need to be tailored to minimise confounding and prevent aggravation of symptoms due to migraine sensitisation. Future research into cervical musculoskeletal impairments or cervical interventions for the migraine population should differentiate between individuals with and without neck pain of cervical origin. Further research is needed to determine the relevance of cervical dysfunction to migraine.

## Data Availability

Not applicable.
